# Better recognition, diagnosis and management of non-IgE-mediated cow’s milk allergy in infancy: iMAP—an international interpretation of the MAP (Milk Allergy in Primary Care) guideline

**DOI:** 10.1186/s13601-017-0162-y

**Published:** 2017-08-23

**Authors:** Carina Venter, Trevor Brown, Rosan Meyer, Joanne Walsh, Neil Shah, Anna Nowak-Węgrzyn, Tong-Xin Chen, David M. Fleischer, Ralf G. Heine, Michael Levin, Mario C. Vieira, Adam T. Fox

**Affiliations:** 1 Section of Allergy and Immunology, University of Colorado Denver School of Medicine, Children’s Hospital Colorado , Box B518, 13123 East 16th Avenue, Anschutz Medical Campus, Aurora, CO 80045 USA; 2 Children’s Allergy Service, Ulster Hospital, Belfast, BT16 1RH Northern Ireland, UK; 3 Department Paediatrics, Imperial College, London, London, W2 1NY UK; 4 Gurney Surgery, Castle Partnership, 101-103 Magdalen Street, Norwich, NR3 1LN UK; 5 Gastroenterology Department, Great Ormond Street Hospital, London, WC1N 3JH UK; 6 Jaffe Food Allergy Institute, Icahn School of Medicine at Mount Sinai, New York, NY 10029 USA; 7 Department of Allergy and Immunology, Shanghai Children’s Medical Center, Shanghai Jiao Tong University School of Medicine, 1678 Dongfang Road, Shanghai, 200127 China; 8 Royal Children’s Hospital Melbourne, Murdoch Children’s Research Institute, Parkville, VIC 3052 Australia; 9 Division of Paediatric Allergy and Asthma, Red Cross War Memorial Children’s Hospital, University of Cape Town, Room 516, ICH Building, Cape Town, South Africa; 10 Centro de Gastroenterologica Pediatrica, Department of Paediatrics, Hospital Pequeno Principe, Pontificia Universidade Catolica do Parana, Curitiba, Brazil; 11 Department of Paediatric Allergy, Guys and St Thomas’ Hospitals NHS Foundation Trust, London, UK; 12 Division of Asthma, Allergy and Lung Biology, King’s College, London, London, UK

## Abstract

Cow’s milk allergy (CMA) is one of the most common presentations of food allergy seen in early childhood. It is also one of the most complex food allergies, being implicated in IgE-mediated food allergy as well as diverse manifestations of non-IgE-mediated food allergy. For example, gastrointestinal CMA may present as food protein induced enteropathy, enterocolitis or proctocolitis. Concerns regarding the early and timely diagnosis of CMA have been highlighted over the years. In response to these, guideline papers from the United Kingdom (UK), Australia, Europe, the Americas and the World Allergy Organisation have been published. The UK guideline, ‘Diagnosis and management of non-IgE-mediated cow’s milk allergy in infancy—a UK primary care practical guide’ was published in this journal in 2013. This Milk Allergy in Primary Care (MAP) guideline outlines in simple algorithmic form, both the varying presentations of cow’s milk allergy and also focuses on the practical management of the most common presentation, namely mild-to-moderate non-IgE-mediated allergy. Based on the international uptake of the MAP guideline, it became clear that there was a need for practical guidance beyond the UK. Consequently, this paper presents an international interpretation of the MAP guideline to help practitioners in primary care settings around the world. It incorporates further published UK guidance, feedback from UK healthcare professionals and affected families and, importantly, also international guidance and expertise.

**Electronic supplementary material:**

The online version of this article (doi:10.1186/s13601-017-0162-y) contains supplementary material, which is available to authorized users.

## Background

Over the last 2 decades, many countries have seen a significant rise in the number of children suffering from food allergy, defined as an adverse health effect arising from a specific immune response that occurs reproducibly on exposure to a given food [1]. The impact on quality of life for families with food allergy has been shown to be significantly worse than for those with chronic pain disorders [2] or diabetes [3]. For most infants with suspected cow’s milk allergy (CMA) this can be clinically subdivided into either immediate-onset *IgE*-*mediated,* where the adverse effects appear usually within minutes following ingestion or delayed onset *non*-*IgE*-*mediated* where the effects develop usually after ≥2 h [4]. It is difficult to define IgE-mediated food allergy into milder and more severe forms as external factors often determine the severity of reaction, with anaphylaxis being the most severe presentation [5]. The spectrum of non-IgE-mediated CMA is broad; encompassing symptoms that range in severity from mild rectal bleeding in milk protein induced proctocolitis to the severe vomiting and collapse that can be seen in food protein induced enterocolitis syndrome (FPIES). Evidence from the United Kingdom (UK) [6] shows that the majority of infants presenting with suspected CMA fall into a ‘mild-to-moderate’ [7] clinical expression of non-IgE-mediated allergy. Although the severities of the non-IgE-mediated reactions were not clearly defined, data from the EuroPrevall study indicates the presence of milder forms of non-IgE-mediated food allergy in Europe, particularly in the Netherlands, Italy and Poland [8].

Whilst attempting to monitor this overall rise in suspected food allergy in children, some controversy has arisen over the true incidence of this ‘mild-to-moderate’ non-IgE-mediated sub-group presenting characteristically in infancy with mostly gastrointestinal-related symptoms such as abdominal discomfort, gastro-oesophageal reflux and abnormal bowel frequency and consistency. In 2015 Schoemaker et al. [8] reported, as part of the EuroPrevall project that the national incidences of CMA in Europe vary across countries with the majority of children with CMA in the UK and the Netherlands suffering from the non-IgE-mediated form. However, the very low incidences reported in some countries have become the subject of debate. Nowak-Wegrzyn et al. [9] and Koletzko et al. [10] argued that the children with non-IgE-mediated CMA in 4 out of the 9 EuroPrevall countries were selectively missed due to clinical unawareness of gastro-intestinal symptoms and their relation to possible CMA. Non-IgE-mediated food allergy is also often reported in Latin America [11]. In line with the previously published MAP (Milk Allergy in Primary Care) guideline [7], the diagnosis of mild-to-moderate non-IgE-mediated CMA requires the strict avoidance of all cow’s milk containing foods for an agreed trial period, i.e. an elimination diet, followed by clinical improvement and then subsequent relapse coincident with reintroduction. This elimination-reintroduction sequence is the only way of reliably diagnosing gastrointestinal manifestations of non-IgE-mediated CMA in infants such as infantile allergic proctocolitis, mild-to-moderate allergic enteropathy and cow’s milk-induced gastro-oesophageal reflux or constipation because there is no allergy skin or blood test for non-IgE-mediated food allergy.

This paper, whilst acknowledging all the possible clinical presentations of CMA in infancy (IgE and non-IgE with their differing diagnostic approaches), will focus primarily on the better recognition, confirmation and management of these infants presenting with suspected mild-to-moderate non-IgE-mediated CMA. The actual management of IgE-mediated CMA and the more severe presentations of non-IgE-mediated CMA, such as FPIES, Eosinophilic Esophagitis and food protein induced enteropathy with faltering growth will not be addressed. This iMAP guideline builds on the strengths of the previous UK MAP guideline, designed with a UK primary care focus, which has been demonstrated to effectively improve the recognition and earlier diagnosis of mild-to-moderate non-IgE-mediated CMA [12] but has now been reviewed with an international focus. The guideline does not represent the views of, nor is it endorsed by, any professional organisation, nor was it supported by any commercial entity at any point in the development process.

## Considerations behind the publication of the 2013 UK MAP guideline [7]

A UK birth cohort study published in 2008 showed that 2–3% of 1–3 year olds suffer from confirmed CMA [13]. Worldwide this prevalence ranges between 1.9 and 4.9% [14], making it one of the most common food allergies in the first years of life. In 2010 a review of 1000 infants with CMA randomly chosen from a UK primary care database [6] showed that 86% were first diagnosed in primary care and that the majority remained there for their care. 42% of the infants were referred on, usually to the care of a general paediatrician. Only a few were seen at a specialist level multidisciplinary paediatric allergy service. The majority presented clinically with mild-to-moderate symptoms of suspected non-IgE-mediated CMA. Significantly smaller numbers could have been categorised as either severe non-IgE-mediated CMA or immediate-onset IgE-mediated CMA. The review highlighted evidence of under-recognition, misdiagnosis, significant delay in diagnosis and sub-optimal management of the infants especially in choosing the most appropriate initial alternative formula suitable for the management of CMA, when breast milk is not available. Fewer than 1 in 5 families had received support from a dietitian [6]. The problem of over and under diagnosis of CMA with its inherent undesirable nutritional management is not unique to the UK. Van den Hooge et al. [15] and Vieira et al. [11] report similar problems in the Netherlands and Latin America respectively. To address the need for better diagnosis of food allergy, six international guideline papers were published from the: United States (US) [1], World Allergy Organization (WAO) [14], European Academy of Allergy, Asthma and Clinical Immunology (EAACI) [16], UK, National Institute of Health and Care Excellence (NICE) [17], British Society for Allergy and Clinical Immunology (BSACI) [18] and European Society for Paediatric Gastroenterology Hepatology and Nutrition (ESPGHAN) [19].

The UK NICE 2011 clinical guideline on the ‘Diagnosis and assessment of food allergy in children and young people in primary care and community settings’, Clinical Guideline 116 (CG116) [17], addressed within its given scope only the presentation and initial assessment of any suspected food allergy. As part of the initial assessment, it particularly emphasised the need to clinically differentiate between non-IgE-mediated and IgE-mediated expressions of food allergy.

Subsequently, a subgroup of the clinicians on the NICE guideline development group published the MAP guideline in 2013 [7]. It addressed in a simple algorithm-based pathway the initial presentation of the differing clinical expressions of CMA in infancy (both non-IgE and IgE) and the on-going management in primary care of those children with confirmed mild-to-moderate non-IgE-mediated CMA.

## Considerations behind the publication of this 2017 updated version of MAP

Evidence showing the effectiveness of the MAP guideline [7] in positively changing UK prescribing patterns has been published [12]. Since 2013, frequent citations and use of MAP across the world have indicated that it is of practical clinical relevance not only for the UK but also for healthcare professionals working in other national healthcare systems. The important early healthcare contacts where the possibility of CMA needs to be explored between parents and a ‘first contact’ clinician do not essentially change from one healthcare system to another.

Significantly, UK NICE has now produced two further publications; in 2015 a NICE Clinical Knowledge Summary (CKS) on the diagnosis and management in primary care of ‘cow’s milk protein allergy in children’ [20], and in 2016 the NICE Quality Standard for food allergy [21]. Since the publication of the MAP guideline in 2013, the BSACI also published their specialist guidelines on cow’s milk allergy [18]. Since then, to our knowledge no other CMA guidelines have been published internationally.

This growing number of guidelines with clinical relevance to CMA gives rise to the very real potential for ‘guideline overload’. A recent UK paper surveyed over 400 general practitioners (GPs) and 300 parents looking at the current ‘journey from diagnosis to management of milk allergy’ for parents and the doctors in primary care [22]. The authors suggested an ideal pathway for the better identification and management of CMA by healthcare professionals should include improved education focusing on the current guidelines and the development of simple tools from the guidelines, such as algorithms, to aid diagnosis and management. A required action highlighted by the parents was the development of a simple tool centred on their recording of possible symptoms that they could take to the appointment with their healthcare professional. Meeting such requests will be of practical clinical relevance for healthcare professionals and families in all healthcare systems.

These NICE primary care guidelines are UK focused and were not intended to be accessed and interpreted by clinicians based outside of the UK. However, the guidance was widely adopted outside of the UK, suggesting the need for an updated non-UK focused interpretation. The aim of this paper is therefore to both incorporate these recent UK publications and to adapt MAP into a more internationally suited version. Management of Milk Allergy in Primary Care (iMAP), to act as both a UK and international guideline with amended algorithms (Figs. 2, 3), supported by other practical tools for both families and healthcare professionals in primary care (Additional files 1, 2, 3, 4).

Clinicians recognise the important role families and carers have in supporting children with food allergy and that ‘family members and carers should be involved in the decision-making process about investigations, treatment and care’ [20]. This iMAP version aims to facilitate that important role.

## Presentation and recognition of CMA

### Revisiting CMA nomenclature

The UK NICE guidelines along with other national and international guidelines clearly indicate that CMA is broadly divided into IgE-mediated and non-IgE-mediated disease. Although they acknowledge that the non-IgE-mediated presentation can be divided into mild-to-moderate and more severe presentations, there is currently no international consensus with clearly agreed definitions of these presentations. Indeed even in terms of Eosinophilic Oesophagitis (EoE), experts and international bodies disagree about whether this is a disease that is primarily non-IgE-mediated [23] or in fact a mixed IgE and non-IgE-mediated disease [1].

### The allergy-focused clinical history

The allergy-focused clinical history continues to form the ‘cornerstone of diagnosis’ in food allergy and ‘children and young people with suspected food allergy should have an allergy-focused clinical history taken’ [21]. EAACI also recently published a task force report on how to take an allergy-focused diet history to aid with the diagnosis of a food allergy [24].

This process will support the clinician to distinguish between IgE-mediated and non-IgE-mediated reactions, based primarily on the information provided by the family. This will then inform the healthcare professional with the appropriate competencies/clinical expertise to decide which other tests, if any, are needed to confirm the diagnosis and then how the food allergy should be managed.

- Any family history of atopic disease in parents or siblings.
- Any history of early atopic disease in the infant.
- The infant’s feeding history including growth.
- Presenting symptoms and signs that may be indicating possible CMA.
- Details of previous management, including any medication and the perceived response to any treatment or dietary change.

**Fig. 1:**
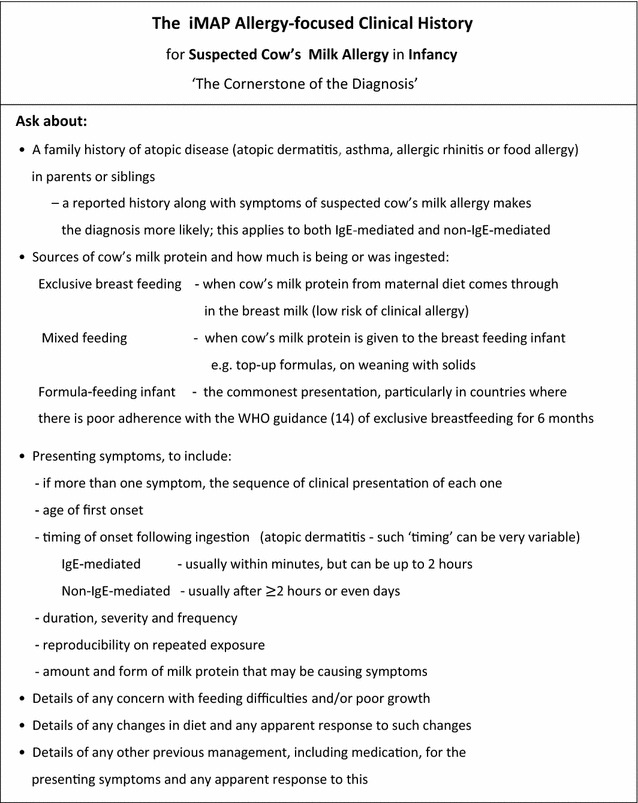
Taking and allergy focussed clinical history: Adapted from the UK NICE guideline CG116 on food allergy

Figure 1 provides a list of questions to ask during the allergy-focused history, but in short such a history will focus on the following questions [7].

**Fig. 2:**
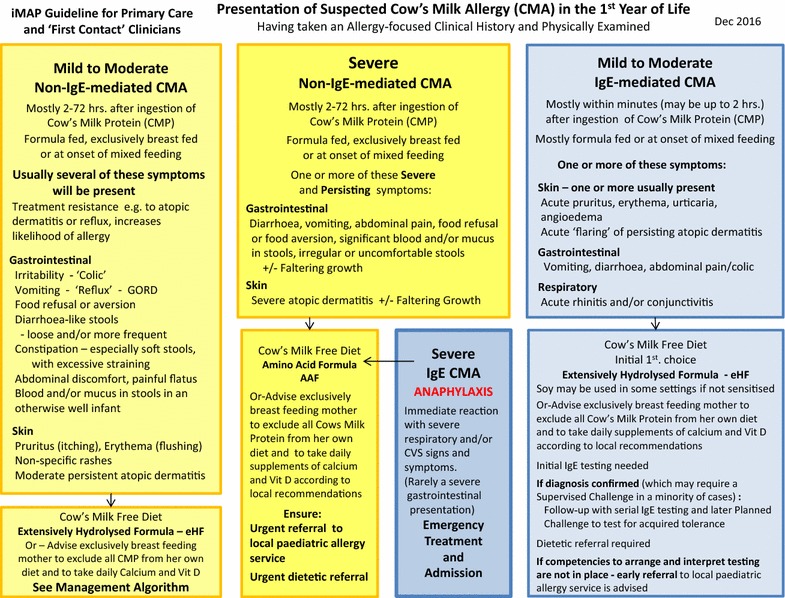
Presentation of suspected cow’s milk allergy (CMA) in the 1st year of Life

The symptoms of the infant at first presentation are a key feature in the diagnostic process. It is important to consider that possible symptoms (Fig. 2) can be variable and overlap with common infant health issues such as irritability (colic), gastro-oesophageal reflux and atopic dermatitis that may not necessarily be CMA-related. There is also often confusion between immediate-onset IgE-mediated allergy and delayed-onset non-IgE-mediated allergy symptoms.

An attempt should therefore be made to elicit a history of all symptoms, assess which are significantly out of the range of normal, and classify them as indicating suspected IgE-mediated or non-IgE-mediated disease. The 4 possible symptom complexes of: IgE-mediated disease (mild-to-moderate or severe) and non-IgE-mediated disease (mild-to-moderate or severe) serve as an entry point for an initial dietary management strategy (Fig. 2).

Recognising the importance of the first contact consultation between the family and the healthcare professional, the iMAP guideline team are in the process of developing a symptom tool. There is currently just one published Cow’s milk related symptom score (CoMiSS) and this needs further validation [25]. Initial data however, indicates that a change in CoMiSS from baseline to month 1 after milk exclusion, can be used to predict CMA [26] (Fig. 2).

As part of the allergy-focused healthcare consultation, it is important to carry out a physical examination [20], particularly looking for signs indicating any allergy-related comorbidities such as atopic dermatitis and, in addition, performing weight, length and head circumference measurements.

## Diagnosis

### Diagnosis of non-IgE-mediated CMA

**Fig. 3:**
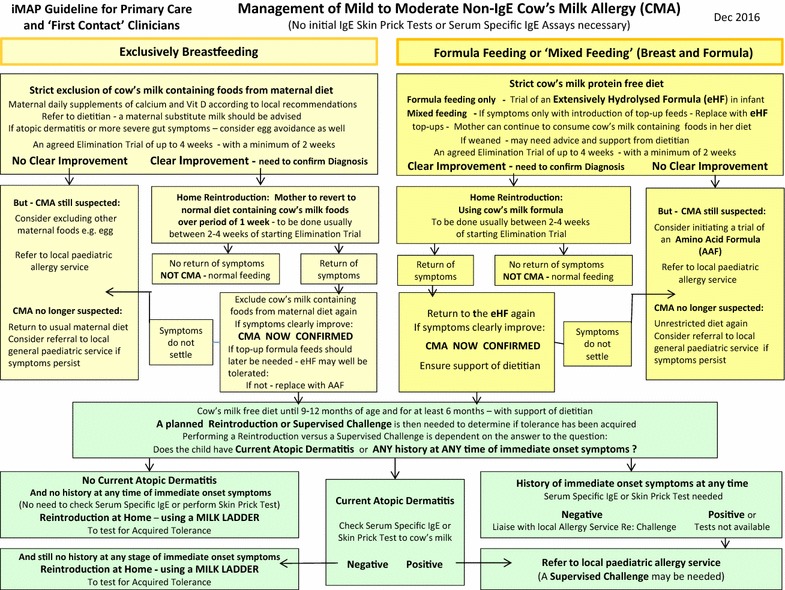
Management of mild to moderate non-IgE cow’s milk allergy (CMA)

If the clinical history suggests non-IgE-mediated CMA and the child ‘has not had a severe delayed reaction’, it is recommended to offer a trial elimination of the suspected allergen and subsequent reintroduction [21] (Fig. 3).

We have previously described indications for the different formulas for the diagnosis and management of the varying expressions of CMA based on a consensus of national and international guidelines [7]. As the iMAP guideline focuses on the diagnosis and management of mild-to-moderate CMA, we wish to highlight here the following points:

Extensively hydrolysed formulas (eHFs) continue to be recommended as the initial prescribed formulas for most infants presenting with suspected mild-to-moderate CMA. However, worldwide, there is at least one example of national practice where an amino acid-based formula (AAF) is commonly chosen as the initial diagnostic trial formula in all suspected cases of CMA, e.g. China. Therefore, the authors acknowledge that practice varies and in some countries an AAF is used as the initial diagnostic trial formula for CMA. It is however important to note that such practice is based on local services, reimbursements of formulas and not born out of clinical evidence based indications for AAF [27].

When an infant reacts to the amount of milk protein passed on from maternal consumption during breastfeeding, it is recommended to avoid cow’s milk from the maternal diet as the first priority. If a supplemental formula is required, an eHF or AAF may be utilised [28]. This decision needs to be taken on an individualised basis, as this depends on whether full symptom resolution has occurred on a maternal elimination diet, the nutritional status of the infant and the underlying CMA diagnosis.

In view of the international focus of the iMAP guidelines, we want to acknowledge that soy formula may be used as the first line alternative to cow’s milk formula in some countries, e.g. South Africa. It is however not recommended under 6 months of age in the USA, Europe, UK, Brazil and Australia and not as the first line of treatment except for Australia where soy may be recommended as first line treatment in infants over 6 months of age in certain CMA conditions [14, 29].

Therefore, local interpretation of the iMAP guidelines may be required in some clinical scenarios.

The reintroduction step, following clear improvement of symptoms during the elimination trial, is of ultimate importance to confirm the diagnosis. In the absence of such a planned reintroduction step to confirm the return of symptoms and their subsequent resolution on recommencing the elimination diet, the risk of a significant number of infants continuing unnecessarily on an expensive and nutritionally demanding diet will remain. The optimum time to explain and agree on the need for the planned early reintroduction is when the trial elimination diet is first started. At that consultation it can be helpful to allow the family to take away with them a factsheet explaining why such a trial elimination diet is needed, followed by a planned reintroduction (Additional file 1).

The iMAP Management Algorithm for mild-to-moderate non-IgE-mediated CMA (Fig. 3) provides guidance on the length and type of the initial elimination diet; for up to 4 weeks (with a minimum of 2 weeks) in conjunction with optimal dietary advice. It is important to emphasise that we are looking for a clear improvement and not necessarily a complete resolution of symptoms [22].

There is an accompanying iMAP written protocol for both the parent and healthcare professional, setting out how cow’s milk protein can then be gradually, simply and safely reintroduced into either the mother’s or infant’s diet at home to confirm or exclude the diagnosis (Additional file 2).

### Diagnosis of IgE-mediated CMA

If the clinical history suggests IgE-mediated CMA, then ‘further testing is recommended’ [21]. This can be done as ‘either a skin prick test or blood test for specific IgE antibodies to the suspected food allergens…’ [21] (Fig. 2).

It is important to recognise that a positive skin prick test or a positive serum specific IgE blood test simply shows sensitisation (i.e. presence of IgE antibodies) to a food allergen, but, on its own, does not confirm an allergy. The final diagnosis of clinical allergy depends on the interpretation of the results in the context of the clinical history and made by a clinician with the appropriate training and skills [21]. The ability of primary care-based clinicians to perform and interpret these tests will differ from country to country. In many cases these tests should ideally be performed in secondary care or allergy referral centers [20]. Additionally, in some cases the history and the allergy test results will not be sufficient to confirm the diagnosis. A supervised food challenge will then be required, and must only be performed under the care of medical providers with the relevant training and skills [17].

It should be emphasised that the iMAP early Home Reintroduction to confirm diagnosis and then the iMAP Home Milk Ladder to test for later acquired tolerance should only be used in children with mild-to-moderate non-IgE-mediated CMA and not in other presentations such as IgE-mediated CMA or severe non-IgE-mediated CMA (e.g. FPIES).

Whilst waiting for a specialist assessment, the iMAP Presentation Algorithm (Fig. 2) guides as to the necessary change to either the maternal diet or infant formula. It may also be helpful to direct parents to national patient support websites.

## Management of mild-to-moderate confirmed non-IgE-mediated CMA within primary care or by the ‘first contact’ clinician (Fig. 3)

When the diagnosis of mild-to-moderate non-IgE-mediated CMA is confirmed, iMAP provides guidance on the on-going management in primary care and recommends dietetic support. The management will include continuation of treatment with a suitable alternative formula or, if indicated, maternal allergen avoidance. Most importantly, milk free weaning advice should be provided by the dietitian not only to ensure that cow’s milk is avoided in the infant’s complementary diet, but also to address growth [11], nutritional [30] and feeding problems in the short [31] and long term [32].

The on-going management includes a second planned reintroduction of milk protein when the time comes to test for acquired tolerance. The iMAP Management Algorithm guides on the timing of this as well. It is usually carried out in the form of a graduated ‘Milk Ladder’ (Additional file 3). Ideally at this stage a dietitian will be taking the lead.

In the development of this new iMAP Milk Ladder, a number of factors were taken into account such as the dose of cow’s milk protein provided, the timing and temperature of heating, as well as the matrix effect of wheat and fat [5]. Healthy eating, general feeding practices across the world and other food allergies have also been taken into account. The dietitians involved in developing the Ladder have therefore reduced the number of Steps in the Ladder. Many of the high sugar foods have been removed as a necessary step, but are still offered as an option, once a certain Step has been passed, e.g. milk chocolate/candy can be given once Step 5 (yoghurt) is passed. Foods eaten by only certain cultural groups have been removed, such as Shepherd’s pie.

## Suspected severe non-IgE mediated CMA

In the uncommon situation of the infant presenting with more severe symptoms leading to a suspected severe expression of non-IgE-mediated CMA, the iMAP Presentation Algorithm (Fig. 2) identifies these infants, advises on the need for early onward referral to a specialist allergy service. For most of these infants the severity of the symptoms and their significant improvement on commencing the elimination diet will be enough to confirm the diagnosis. However, should they still need an early food challenge to confirm or exclude the diagnosis that will need to be done under the careful supervision of a specialist allergy team [21].

## Referral

Any specialist allergy service for children should be led by a paediatrician or other appropriately trained physician, supported by a multidisciplinary team made up of specialist dietitians, nurses and ideally a clinical psychologist all with the necessary expertise in childhood food allergy. Due to the multisystem involvement of CMA, other medical specialities may also need to be readily accessible, including gastroenterology and dermatology.

## Conclusion

CMA is one of the most common food allergies affecting children worldwide and, with few exceptions, presents in the first months of life. Clinically it is complex due to its differing possible presentations making it challenging to diagnose. These factors have underscored the need for UK NICE guidance as well as the MAP primary care CMA guideline written by a group of the clinicians who were part of the NICE food allergy guideline development group. Most infants who present with suspected CMA have non-IgE-mediated manifestations with mild-to-moderate and delayed-onset symptoms. However, those infants who present with either immediate-onset IgE-mediated symptoms or those who progress to more severe non-IgE-mediated symptoms need to be promptly and reliably identified in order to allow early referral.

There is evidence that there are significant care issues for these young infants with any expression of CMA and also for their families. Evidence showing the effectiveness of the earlier MAP guideline in positively changing UK prescribing patterns has been published. This newer MAP guideline version, iMAP (based now on a wider range of relevant UK NICE publications and the input of international clinical experts) with its amended algorithms and growing portfolio of accompanying practical tools should help both primary care or ‘first contact’ clinicians work better with the families to further improve the quality of care. Hopefully this will lead to better health outcomes including: the earlier identification of CMA, early referral of children requiring specialist allergy review and identification of those remaining children who can be effectively and safely managed in primary care. This approach will be of practical clinical relevance not only for the UK but also for healthcare professionals working in other national healthcare systems.

## Additional files

**Additional file 1.** The iMAP Initial Factsheet for Parents.

**Additional file 2.** The iMAP Home Reintroduction Protocol to Confirm Diagnosis.

**Additional file 3.** The iMAP Milk Ladder.

**Additional file 4.** The Recipes.

## Abbreviations

MAP: Milk Allergy in Primary Care
CMA: cow’s milk allergy
FPIES: food protein induced enterocolitis syndrome
UK: United Kingdom
US: United States
WAO: World Allergy Organization
EAACI: European Academy of Allergy, Asthma and Clinical Immunology
NICE: National Institute of Health and Care Excellence
BSACI: British Society for Allergy and Clinical Immunology
ESPGHAN: European Society for Paediatric Gastroenterology Hepatology and Nutrition
CG116: Clinical Guideline 116
CKS: NICE Clinical Knowledge Summary
GPs: general practitioners
iMAP: International version: Management of Milk Allergy in Primary Care
CoMISS: cow’s milk related symptom score
eHFs: extensively hydrolysed formulas
AAF: amino acid-based formula
