# Correction to: Better recognition, diagnosis and management of non-IgE-mediated cow’s milk allergy in infancy: iMAP—an international interpretation of the MAP (Milk Allergy in Primary Care) guideline

**DOI:** 10.1186/s13601-017-0189-0

**Published:** 2018-01-25

**Authors:** Carina Venter, Trevor Brown, Rosan Meyer, Joanne Walsh, Neil Shah, Anna Nowak-Węgrzyn, Tong-Xin Chen, David M. Fleischer, Ralf G. Heine, Michael Levin, Mario C. Vieira, Adam T. Fox

**Affiliations:** 1 Section of Allergy and Immunology, University of Colorado Denver School of Medicine, Children’s Hospital Colorado, Box B518, 13123 East 16th Avenue, Anschutz Medical Campus, Aurora, CO 80045 USA; 2 Children’s Allergy Service, Ulster Hospital, Belfast, BT16 1RH Northern Ireland, UK; 3 Department Paediatrics, Imperial College, London, London, W2 1NY UK; 4 Gurney Surgery, Castle Partnership, 101-103 Magdalen Street, Norwich, NR3 1LN UK; 5 Gastroenterology Department, Great Ormond Street Hospital, London, WC1N 3JH UK; 6 Jaffe Food Allergy Institute, Icahn School of Medicine at Mount Sinai, New York, NY 10029 USA; 7 Department of Allergy and Immunology, Shanghai Children’s Medical Center, Shanghai Jiao Tong University School of Medicine, 1678 Dongfang Road, Shanghai, 200127 China; 8 Royal Children’s Hospital Melbourne, Murdoch Children’s Research Institute, Parkville, VIC 3052 Australia; 9 Division of Paediatric Allergy and Asthma, Red Cross War Memorial Children’s Hospital, University of Cape Town, Room 516, ICH Building, Cape Town, South Africa; 10 Centro de Gastroenterologica Pediatrica, Department of Paediatrics, Hospital Pequeno Principe, Pontificia Universidade Catolica do Parana, Curitiba, Brazil; 11 Department of Paediatric Allergy, Guys and St Thomas’ Hospitals NHS Foundation Trust, London, UK; 12 Division of Asthma, Allergy and Lung Biology, King’s College, London, London, UK

## Correction to: Clin Transl Allergy (2017) 7:26 10.1186/s13601-017-0162-y

In the original version of this article [1], published on 23 August 2017, an incorrect version of Additional file 4 has been used. The corrected version of Additional file 4 is given in this correction.

## Additional file

**Additional file 4.** The Recipes.
