# Supplementary material for: Better recognition, diagnosis and management of non-IgE-mediated cow’s milk allergy in infancy: iMAP—an international interpretation of the MAP (Milk Allergy in Primary Care) guideline
Source: Clin Transl Allergy. 2017 Aug 23;7:26. doi: 10.1186/s13601-017-0162-y (PMC5567723; doi:10.1186/s13601-017-0162-y)
Supplement: Supplementary file 1 — Additional file 1. The iMAP Initial Factsheet for Parents. [file 13601_2017_162_MOESM1_ESM.pdf]

The iMAP Milk Allergy Guideline
– Initial Fact Sheet for Parents

iMAP GUIDELINE

When Cow's Milk Allergy is being considered as a possible cause of your baby's symptoms

Food Allergy has become more common and happens when a child's Immune System wrongly reacts against some of the proteins in a food, thinking they may be harmful. This will then result in either the onset of an immediate allergic reaction or a more delayed onset allergic reaction.

You have been given this factsheet because your baby may now be showing mild-to-moderate symptoms of a delayed type of allergic reaction to the proteins in cow's milk.

The **typical mild-to-moderate** symptoms include one **or usually more than one** of the following:

- **Irritability (colic), reflux, vomiting, refusing or disliking being fed, loose or frequent stools, constipation (especially straining to pass even a soft stool), pain in the tummy, a little blood or slime in the stools**
- **Itching of the skin, redness of the skin, a tendency to 'rashes', atopic dermatitis/eczema (dry inflamed itchy patches of skin)**

Delayed Onset Cow's Milk Allergy

Correct up-to-date medical term is
Non-IgE Cow's Milk Allergy
(This is still often incorrectly referred to as Cow's Milk Protein Intolerance)

The Immune System gradually builds up this mistaken reaction and as the milk protein continues to be consumed,it may result in a pattern of symptoms developing over hours, days or even weeks. It can occur with breast feeding alone (exclusive breast feeding) due to the small amount of cow's milk protein that passes across into the breast milk when the mother herself consumes cow's milk or dairy products but is more likely to occur later in such a breast fed baby when the time comes for formula, dairy products (e.g. yoghurt) or cow's milk to be added into the baby's own diet (mixed feeding). However, it is much more likely to occur in the baby who is just being bottle-fed.

Cow's milk allergy is more likely to be the cause of these symptoms if there is a history of atopic dermatitis/eczema, asthma, hay fever or food allergy in any close family members: mother, father, brothers or sisters. However such a history does not have to be present.

There are no skin or blood tests for delayed onset cow's milk allergy (Non-IgE allergy). The only reliable test is to take all the cow's milk protein out of the diet of the exclusively breast feeding mother or out of the diet of the bottle fed infant and then to later reintroduce it in a planned way.

It can be difficult to identify when some of these symptoms (e.g. irritability/colic, reflux/vomiting, and feeding difficulties), which so often occur in babies, are actually due to cow's milk allergy. In most cases allergy will not be the cause and each of the symptoms will settle in time, most needing minimal if any treatment. The **iMAP guideline** is designed to initially help your healthcare professional identify which babies should be suspected of having cow's milk allergy by listing these possible symptoms. The guideline then goes on to show how the diagnosis can be confirmed and then how to manage those babies with mild-to-moderate delayed onset cow's milk allergy (Non-IgE allergy).

All cow's milk and cow's milk containing foods must be removed from your diet if exclusively breast feeding or from your baby's diet **for a period of up to 4 weeks (minimum 2 weeks)**, as this is the time it may take for the symptoms to improve. Your healthcare professional will advise how your diet will need to change if you are exclusively breast feeding or will prescribe a special low allergy formula for your baby if he/she is being bottle fed. During the trial, the symptoms will either begin to clearly improve, suggesting the presence of cow's milk allergy, or there will be no significant change, which usually excludes the diagnosis of allergy.

At the end of this agreed trial it is really important to reintroduce the cow's milk protein.

If exclusively breast feeding you can simply start eating dairy products again or if your baby is bottle fed the previous cow's milk based formula can be reintroduced. A further **iMAP Parents Guide** will provide you with details of how to do this gradually, safely and easily at home. This will show whether any clear improvement seen in symptoms during the trial was actually due to cow's milk allergy and not just to your baby improving naturally. If allergy is the cause, the symptoms can be expected to return within the first few days of reintroducing the milk protein, but will usually settle well again as the milk free diet is restarted.

By following this guide, **the diagnosis of cow's milk allergy can then be properly confirmed or excluded**. If the diagnosis is confirmed, the next steps in management will be explained to you and they should importantly include the on-going support of a dietitian.

There are other types of adverse reactions to milk

Immediate Onset Cow's Milk Allergy
and **Lactose Intolerance**

There is often confusion around how these two conditions differ from Delayed Onset Milk Allergy.

Immediate Onset Cow's Milk Allergy

(Correct medical term is IgE Cow's Milk Allergy)
This immediate onset type of food allergy usually affects slightly older children and is often due to other foods such as egg, peanut, tree nuts or sea food. It can however occur with cow's milk, most commonly when cow's milk based formula feeds are used as 'top-ups' in the breast fed baby or later when weaning with solids begins and dairy products (e.g. yoghurt) or even cow's milk are added into the diet of these babies. **Symptoms usually develop within minutes following ingestion.** They may be mild, such as reddening of the skin, hives, and puffiness around the mouth or eyes. Rarely much more serious 'internal' symptoms can develop; especially breathing difficulties (this is called Anaphylaxis).

Lactose Intolerance

The sugar in cow's milk and in breast milk is called lactose. An enzyme called lactase, present in the gut is needed to break this complicated sugar into smaller sugars that the body can then absorb and use. As young children grow up and drink less milk, the amount of this enzyme gradually and naturally falls. For some children, especially those from an Asian or African ethnic background, this may mean that over time not all the lactose they consume is broken down. Very gradually gut symptoms begin to develop. These symptoms may include bloating, tummy pains, wind and very loose stools – it does not include constipation or reflux/vomiting. **However this does not usually happen until later in childhood and certainly it very rarely occurs in young babies.**
