# Supplementary material for: Better recognition, diagnosis and management of non-IgE-mediated cow’s milk allergy in infancy: iMAP—an international interpretation of the MAP (Milk Allergy in Primary Care) guideline
Source: Clin Transl Allergy. 2017 Aug 23;7:26. doi: 10.1186/s13601-017-0162-y (PMC5567723; doi:10.1186/s13601-017-0162-y)
Supplement: Supplementary file 2 — Additional file 2. The iMAP Home Reintroduction Protocol to Confirm Diagnosis. [file 13601_2017_162_MOESM2_ESM.pdf]

The Early Home Reintroduction to Confirm the Diagnosis of Cow's Milk Allergy

iMAP GUIDELINE

Practical Pointers for Parents/ Carers on how to carry out the:

iMAP Home Reintroduction to **Confirm** or **Exclude** the Diagnosis of Mild-to-Moderate Non-IgE Cow's Milk Allergy

After an agreed period of cow's milk protein exclusion has resulted in a clear improvement in symptoms

A carefully planned home reintroduction of cow's milk protein is still needed to either confirm or exclude the diagnosis of cow's milk allergy because any clear improvement in your baby's symptoms could be due to other factors.

1. DO NOT start the Reintroduction if your child is unwell: e.g. Any respiratory or breathing problems (this includes a common cold)
   Any tummy or bowel symptoms
   Any 'teething' symptoms which are thought to be unsettling your child
   If your child has atopic dermatitis/eczema - any current flare-up of the skin

2. DO NOT start the Reintroduction if your child is receiving any medication that may upset the bowels, such as a course of antibiotics

3. DO NOT stop any medication that your baby may be on, e.g. reflux medicine

4. DO NOT introduce any other new foods during the Reintroduction.

5. Keep a record of what your child eats and drinks during the reintroduction and record any possible symptoms such as, vomiting, bowel changes, rashes or changes in their eczema

The Home Reintroduction

How you carry out the Reintroduction depends on whether you are giving any formula milk or are fully breast feeding.

Formula Fed Child

(those taking only formula feeds or taking formula as well as breast feeds)

Each day gradually increase the amount of cow's milk formula only in the FIRST bottle of the day (as set out in the example below). For the rest of the day, all the remaining bottles will continue to be made up only with the special low allergy (hypoallergenic) formula. If you are also breast feeding and on a milk free diet yourself, start eating products containing milk again, e.g milk, cheese and yoghurt.

If the symptoms return, **STOP** the Reintroduction. Give only the prescribed formula again and inform your doctor or dietitian. Your child's symptoms should settle again within a few days and the diagnosis of cow's milk allergy is now confirmed.

If no symptoms occur after day 7, when you have replaced the 1st bottle of the day completely with cow's milk formula, give your child cow's mik formula in all bottles

If no symptoms occur within 2 weeks of your child having more than 200mls. (almost 7 fl. oz.) of cow's milk formula per day, your child does not have cow's milk allergy.

A Practical Example of a Reintroduction in a Formula Fed Child

**The Days**	**Volume of Boiled Water** mls. (fl. oz.)	**Hypoallergenic Formula** mls. (fl. oz.)	**Cow's Milk Formula** mls. (fl. oz.)
**Day 1**	210 mls. (7 fl.oz.)	180 mls. (6 fl.oz.) in 1st bottle **only**	30 mls. (1 fl.oz.) in 1st bottle **only**
**Day 2**	210 mls. (7 fl.oz.)	150 mls. (5 fl.oz.) in 1st bottle	60 mls. (2 fl.oz.) in 1st bottle
**Day 3**	210 mls. (7 fl.oz.)	120 mls. (4 fl.oz.) in 1st bottle	90 mls. (3 fl.oz.) in 1st bottle
**Day 4**	210 mls. (7 fl.oz.)	90 mls. (3 fl.oz.) in 1st bottle.	120 mls. (4 fl.oz.) in 1st bottle
**Day 5**	210 mls. (7 fl.oz.)	60 mls. (2 fl.oz.) in 1st bottle	150 mls. (5 fl.oz.) in 1st bottle
**Day 6**	210 mls. (7 fl.oz.)	30 mls. (1 fl.oz.) in 1st bottle	180 mls. (6 fl.oz.) in 1st bottle
**Day 7**	210 mls. (7 fl.oz.)	0	210 mls. (7 fl.oz.) in 1st bottle
If no symptoms occur after Day 7, when you have replaced the 1st bottle of the day completely with cow's milk formula, give your child cow's mik formula in all bottles.			

Fully Breast Fed Child

Simply reintroduce cow's milk and cow's milk containing foods into your own diet in amounts previously consumed over a 1 week period. You do not need to do this gradually.

If the symptoms return, **STOP** the Reintroduction, return to your full milk exclusion diet and inform your doctor or dietitian. Your child's symptoms should settle again within a few days and the diagnosis of cow's milk allergy is now confirmed.

If no symptoms occur, you can continue to drink cow's milk and eat cow's milk containing products, e.g. cheese and yoghurt. Your child does not have cow's milk allergy.

In a few children possible symptoms of cow's milk allergy may appear later when larger amounts of cow's milk protein come to be introduced into the child's diet, either when formula milk is introduced or on weaning when milk containing products or plain milk is introduced. Should this happen contact your doctor or dietitian.

Adapted from: Clinical and Translational Allergy 2013, 3:23
