# Supplementary material for: Better recognition, diagnosis and management of non-IgE-mediated cow’s milk allergy in infancy: iMAP—an international interpretation of the MAP (Milk Allergy in Primary Care) guideline
Source: Clin Transl Allergy. 2017 Aug 23;7:26. doi: 10.1186/s13601-017-0162-y (PMC5567723; doi:10.1186/s13601-017-0162-y)
Supplement: Supplementary file 3 — Additional file 3. The iMAP Milk Ladder. [file 13601_2017_162_MOESM3_ESM.pdf]

# THE iMAP MILK LADDER

**To be used only in children with Mild to Moderate Non-IgE Cow's Milk Allergy**
**Under the supervision of a healthcare professional**
PLEASE SEE THE ACCOMPANYING RECIPE INFORMATION

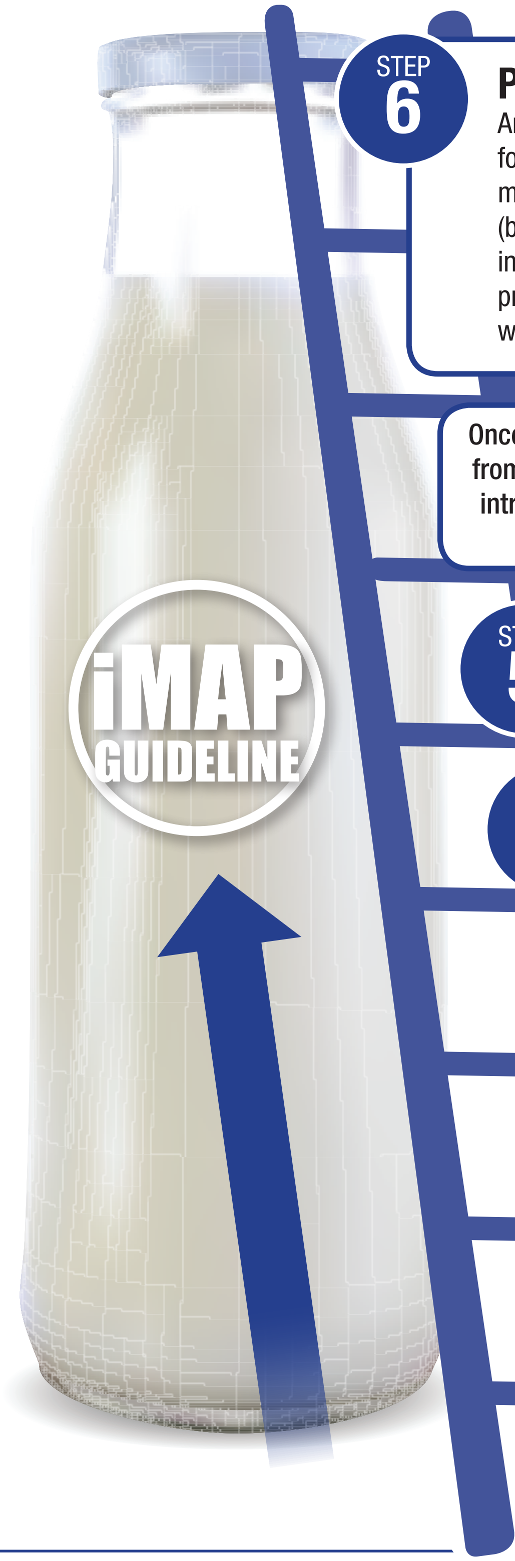


## STEP 6 – Pasteurised milk/suitable infant formula

Amount – start with 100ml (3.5 fl oz) of pasteurised milk/infant formula and mix this with current milk replacement. Build up to 200 ml (7 fl oz). If this is tolerated switch all current milk replacements (bottle and in breakfast cereals) to pasteurised milk or suitable infant formula. Discuss what is an appropriate amount of milk/milk products with your healthcare professional. UHT and sterilised milk will be tolerated as well.

Once your child tolerates yoghurt, butter, spread, chocolate buttons, fromage frais, petit filous (be careful of the sugar content), you can introduce softer cheese like cream cheese and camembert/brie – remember to use pasteurised soft cheese for children

## STEP 5 – Yoghurt

Amount - 125mls (4.5 fl oz)

## STEP 4 – Cheese

Amount 15g (½ fl oz) (hard cheese e.g. cheddar or parmesan) Once your child tolerates cheese, you can introduce 15g baked cheese on a pizza or baked on other food as well.

## STEP 3 – Pancake

Amount - ½ and build up to 1 (see Recipe)

## STEP 2 – Muffin

Amount - ½ and build up to 1 (see Recipe)

## STEP 1 – Cookie/Biscuit

Amount - 1 and build up to 3 (see Recipe)

---

**AT EACH OF THE FOLLOWING STEPS**
**Cookie, muffin, pancake, cheese and yoghurt**
It may be advisable in some cases to start with a ¼ or a ½ of that particular food and then over a few days to gradually build up to a whole portion - Please ask your healthcare professional for guidance on this

**THE LOWER STEPS ARE DESIGNED TO BE USED WITH HOME MADE RECIPES. THIS IS TO ENSURE THAT EACH STEP HAS THE APPROPRIATE MILK INTAKE. THE RECIPES WILL BE PROVIDED BY YOUR HEALTHCARE PROFESSIONAL**
Should you wish to consider locally available store-bought alternatives - seek the advice of your healthcare professional Re: availability

October 2016

# Practical Pointers for Parents/Carers on using at home the iMAP Milk Ladder

iMAP GUIDELINE

**ONLY FOR CHILDREN WHO ARE BEING MANAGED AS MILD-TO-MODERATE NON-IgE COW'S MILK ALLERGY**

The practical concept of this Ladder is the recognised fact that the more 'baked' cow's milk protein is, usually the less allergenic it is. Therefore you will see that Step 1 begins with a form of very well baked milk protein and then the further Steps give examples of gradually less well baked milk protein products.

The following 'Pointers' should make it easier for you to understand how best to use this Ladder. We advise that you are supported by a Healthcare Professional (HCP) until the Ladder has been successfully climbed. This may be your doctor, nurse but ideally your dietitian.

- Before starting the Ladder and progressing to each further Step, please ensure that your child is well at the time and also that any tummy symptoms, bowel symptoms or eczema are settled.
- Most children will start on Step 1. However some may be already eating one or more foods on the Ladder. If that is the case, you need to be advised which Step you should start on.
- The Ladder has 6 Steps, but your HCP may adjust the number of Steps to suit your child best.
- The time spent on each Step will vary from one child to another depending on their individual expression of milk allergy. This should also be discussed and agreed with you.
- The amounts in the Ladder are given as a guide – occasionally smaller or larger amounts may be recommended.
- Each of the early Steps of the Ladder importantly is accompanied by the appropriate recipe (see recipes).
- Each of the recipes has an egg and wheat free option (they are all soya free) to make the Ladder suitable for children who may have other co-existing food allergies.
- If the food on any Step of the Ladder is tolerated, your child should continue to consume this (as well as all the foods in the previous Steps) and then try the food on the next agreed Step.
- If your child does not tolerate the food in a particular Step, simply go back to the previous Step. You should then be advised when that further Step can be tried again.
