# Supplementary material for: Better recognition, diagnosis and management of non-IgE-mediated cow’s milk allergy in infancy: iMAP—an international interpretation of the MAP (Milk Allergy in Primary Care) guideline
Source: Clin Transl Allergy. 2017 Aug 23;7:26. doi: 10.1186/s13601-017-0162-y (PMC5567723; doi:10.1186/s13601-017-0162-y)
Supplement: Supplementary file 4 — Additional file 4. The Recipes. [file 13601_2017_162_MOESM4_ESM.docx]

| **Additional File 4 iMAP Milk Ladder Recipes** | | | | | | | |
| --- | --- | --- | --- | --- | --- | --- | --- |
| **STEP 1: COOKIE OR BISCUIT – SWEET/PLAIN** | | | | | | | |
| **Recipe** | **tsp/tbsp/**  **cup** | **g/ml** | **oz/lb** | **Ingredient** | **Milk per portion (ml)** | **Milk protein per**  **portion** | **Temperature** |
| *1 cookie/biscuit* | | | | | *1 ml* | *0.35 g* |  |
| **Bakes 20 small finger size biscuits/cookies** | 1 cup | 125 g | 4.5 oz | Flour  (wheat or wheat free) |  | | 180⁰C or 350⁰F  Bake for 10 – 15 min  Depending on size of  cookie/biscuit  Practical tip – let dough cool in fridge for 30 min – which makes it easier handle |
|  | ¼ teaspoon | 1 g |  | Xanthan gum – if wheat free flour is used |  |  |  |
|  | ¼ cup | 50 g | 1 ¾ oz | Cold dairy free spread |  |  |  |
|  | ¼-⅓ cup  (may vary depending on fruit) |  |  | Grated apple/pear/  pureed banana |  |  |  |
|  | 1 teaspoon | 2 g |  | Skimmed/non-fat milk  powder* |  |  |  |
|  | Tip of a knife |  |  | Vanilla powder |  |  |  |
| 1. Mix the flour, xanthan gum and milk powder 2. Rub in the cold dairy free spread 3. Mix in the fruit (you may need to add a little bit more if it is too dry) and vanilla powder 4. Roll out and cut in finger sized strips 5. Bake in the oven | | | | | | | |
| Start with 1 cookie/biscuit (equivalent to 1 ml of milk); increase to 2 and then  *3 cookies/biscuits* (equivalent to 3 ml of milk - if child can manage a portion of 3 biscuits/cookies) | | | | | *3 ml* | *0.105 g* | 180⁰C or 350⁰F |

| **STEP 1: COOKIE OR BISCUIT – SAVOURY** | | | | | | | | | | | | | | | |
| --- | --- | --- | --- | --- | --- | --- | --- | --- | --- | --- | --- | --- | --- | --- | --- |
| **Recipe** | **tsp/tbsp/**  **cup** | | | **g/ml** | | **oz/lb** | | **Ingredient** | | **Milk per**  **portion**  **(ml)** | **Milk protein per**  **portion** | | | | **Temperature** |
| *1 cookie/biscuit* | | | | | | | | | | *1 ml* | *0.035g* | | |  | |
| **Bakes 20 small finger size biscuits/cookies** | | 1 cup | 125 g | | 4 ½ oz | | Flour  (wheat or wheat free) | | |  | | | 180⁰C or 350⁰F  Bake for  10 – 15 min  depending on size of cookie/biscuit  Practical tip – let dough cool in fridge for 30 min – which makes it easier handle | | |
|  |  | ¼ teaspoon | 1g | |  | | Xanthan gum – if wheat free flour is used | | |  |  |  |  |  |  |
|  |  | ¼ cup | 50 g | | 1 ¾ oz | | Cold dairy free spread | | |  |  |  |  |  |  |
|  |  | ⅓ cup | 40 g | | 1 ¼ oz | | Grated DAIRY FREE CHEESE | | |  |  |  |  |  |  |
|  |  | 1  teaspoon | 2 g | |  | | Skimmed/non-fat milk powder* | | |  |  |  |  |  |  |
|  |  | 2 tablespoons | 10 ml | |  | | Water | | |  |  |  |  |  |  |
| 1. Mix the flour, xanthan gum and milk powder 2. Rub in the cold dairy free spread 3. Mix in the grated DAIRY FREE CHEESE. Add water (you can add a bit more if it is too dry) 4. Roll out and cut in finger sized strips 5. Bake in the oven | | | | | | | | | | | | | | | |
| Start with 1 cookie/biscuit (equivalent to 1 ml of milk); increase to 2 and then  *3 cookies/biscuits* (equivalent to 3 mls of milk - if child can manage a portion of 3 biscuits/cookies) | | | | | | | | | *3 ml* | | | *0.105 g* | 180⁰C or 350⁰F | | |

| **STEP 2: MUFFIN – SWEET/PLAIN** | | | | | | | | | | |
| --- | --- | --- | --- | --- | --- | --- | --- | --- | --- | --- |
| **Recipe** | **tsp/tbsp/**  **cup** | **g/ml** | **oz/lb** | | **Ingredient** | **Milk per portion (ml)** | | **Milk protein per**  **portion** | | **Temperature** |
| Start with half a muffin and then one muffin: *half a muffin*  *: one muffin* | | | | | | *12.5 ml*  *25 ml* | *0.0.875 g*  *0.875 g* | |  | |
| **Bakes 6 muffins** | 2 cups | 250 g | | 8 oz | Flour  (wheat or wheat free) |  | | | 180⁰C - 200⁰C  or  350⁰F- 400⁰F  Bake for 15 – 20 mins  Tip – use a whisk and milk and oil together. This makes the muffins light | |
|  | ½ tsp | 3 g | |  | Xanthan Gum – if wheat free flour is used |  |  |  |  |  |
|  | 2 ½ tsp | 10 g | | 1/3 oz | Baking powder |  |  |  |  |  |
|  | 2 level  tbsp | 25 g | | 2/3 oz | Sugar – if your child is older you can add 2-3 tablespoons |  |  |  |  |  |
|  | Pinch |  | |  | Salt |  |  |  |  |  |
|  | ¼ cup | 50 ml | | 1 2/3  fl oz | Sunflower oil  or Canola oil |  |  |  |  |  |
|  | 1 cup | 250 ml | | 8 fl oz | Milk** |  |  |  |  |  |
|  | ½ cup  and  1 tbsp | 110 g | | 3.9 oz | Finely chopped/mashed fruit: apple/pear/banana |  |  |  |  |  |
|  | Vanilla essence to taste | | | | |  |  |  |  |  |
| 1. Mix flour, xanthan gum, baking powder, sugar and salt 2. Mix oil and milk together and to the dry ingredients 3. Finally add in chopped fruit and vanilla (additional) and mix through 4. Bake in oven | | | | | | | | | | |
| **1.5 muffin = equivalent to baked milk muffin from Mount Sinai Recipe(1) (which contains 1.3 g milk)** | | | | | | | | | | |

| **STEP 2: MUFFIN – SAVOURY** | | | | | | | | | | | | | |
| --- | --- | --- | --- | --- | --- | --- | --- | --- | --- | --- | --- | --- | --- |
| **Recipe** | **tsp/tbsp/**  **cup** | **g/ml** | | **oz/lb** | | **Ingredient** | | **Milk per portion (ml)** | | | **Milk protein**  **per**  **portion** | **Temperature** | |
| *Start with half a muffin and then one muffin: half a muffin*  *: one muffin* | | | | | | | | | *12.5 ml*  *25 ml* | *0.0.875 g 0.875 g* | | |  |
| **Bakes 6 muffins** | 2 cups | | 250 g | | 8 oz | | Flour  (wheat or wheat free) | |  | | | | 180⁰C - 200⁰C  or  350⁰F- 400⁰F  Bake for 15 – 20 mins  Tip – use a whisk and milk and oil together. This makes the muffins light |
|  | ½ teaspoon | | 3 g | |  | | Xanthan Gum – if wheat free flour is used | |  |  |  |  |  |
|  | 2 ½ tsp | | 10 g | | 1/3 oz | | Baking powder | |  |  |  |  |  |
|  | Pinch | |  | |  | | Salt | |  |  |  |  |  |
|  | ¼ cup | | 50 ml | | 1 2/3  fl oz | | Sunflower oil or Canola oil | |  |  |  |  |  |
|  | 1 cup | | 250 ml | | 8 fl oz | | Milk ** | |  |  |  |  |  |
|  | ½ cup | | 60 g | | 2 oz | | Grated DAIRY FREE CHEESE | |  |  |  |  |  |
| 1. Mix flour, xanthan gum, baking powder and salt 2. Mix oil and milk together and to the dry ingredients 3. Add DAIRY FREE CHEESE; Add a bit of water if required - Feel free to chop in a handful of spinach to add colour and fibre 4. Bake in oven | | | | | | | | | | | | | |
| **1.5 muffin = equivalent to baked milk muffin from Mount Sinai Hospital(1) (which contains 1.3 g milk)** | | | | | | | | | | | | | |

| **Step 3: PANCAKE** | | | | | | | | | | | | | |
| --- | --- | --- | --- | --- | --- | --- | --- | --- | --- | --- | --- | --- | --- |
| **Recipe** | **tsp/tbsp/**  **cup** | | | **g/ml** | | **oz/lb** | | **Ingredient** | **Milk per portion (ml)** | | **Milk protein per**  **portion** | | **Temperature** |
| *Start with half pancake and then 1 pancake as indicated below: half a pancake*  *: one pancake* | | | | | | | | | | *21 ml*  *42 ml* | | *0.735 g*  *1.47 g* |  |
| **Bakes 6 pancakes** | | 1 cup | 125 g | | 4.5 oz | | Flour (wheat or wheat free) | | |  | | | Fry in a hot pan using oil of choice until golden brown and crispy |
|  |  | 2 ½ tsp | 10 g | | 1/3 oz | | Baking powder | | |  |  |  |  |
|  |  | ¼ tsp | 1-2 g | | pinch | | Salt | | |  |  |  |  |
|  |  | 2 tbsp | 30 ml | | 1 fl oz | | Sunflower  or Canola oil | | |  |  |  |  |
|  |  | 1 cup | 250 ml | | 8 fl oz | | Milk** | | |  |  |  |  |
|  |  | 2/3 cup | 50 ml | | 1.5 fl oz | | Water | | |  |  |  |  |
| 1. Add all ingredients into a mixing bowl and mix together 2. Fry in a hot pan | | | | | | | | | | | | | |
| Some children do not like cake or pancake textures: For these children there is the option of boiling a small potato, adding 42 ml of milk  (1.3 g protein) and some milk free spread, cover with foil and bake in the oven for 40 minutes at 180⁰C - 200⁰C  or 350⁰F- 400⁰F. This product does not contain any wheat in the food matrix and it may therefore affect the allergenicity. | | | | | | | | | | | | | |

| **Step 4: CHEESE** | | | | | | | |
| --- | --- | --- | --- | --- | --- | --- | --- |
| **Food** | **tsp/tbsp/**  **cup** | **g/ml** | **oz/lb** | **Ingredient** | **Milk**  **per portion**  **(ml)** | **Milk protein per**  **portion** | **Temperature** |
| **Cheese** | 2 ½ tbsp | 15 g | ½ oz | Cheese*** | *15 g* | *3.43 g* | 85⁰C or 185⁰F (no need to further heat this – just an indication of how cheese is made) |

| **Step 5: YOGHURT** | | | | | | | |
| --- | --- | --- | --- | --- | --- | --- | --- |
| **Food** | **tsp/tbsp/**  **cup** | **g/ml** | **oz/lb** | **Ingredient** | **Milk**  **per portion**  **(ml)** | **Milk protein per**  **portion** | **Temperature** |
| **Yoghurt** | ½ cup | 125 ml | 4.5 fl oz | Yoghurt | 125 ml | 6.0 g  depending on brand | 98⁰C (no need to further heat this – just an indication of how yoghurt is made) |
| Once your child tolerates yoghurt, butter, spread, chocolate buttons, fromage frais, petit filous (be careful of the sugar content), you can introduce softer cheese like cream cheese and camembert/brie – remember to use pasteurised soft cheese for children | | | | | | | |
|  | | | | | | | |
| **Step 6: MILK** | | | | | | | |
| **Food** | **tsp/tbsp/**  **cup** | **g/ml** | **oz/lb** | **Ingredient** | **Milk**  **per portion**  **(ml)** | **Milk protein per**  **portion** | **Temperature** |
| **Pasteurised Milk** | ⅖ cup | 100 ml | 3.5 fl oz | Pasteurised Milk** | *100 ml* | *3.47 g* | 57-68⁰C  15-20 seconds  Both pasteurised milk and infant formula is produced this way – there is no need to further heat this. |
|  | ¾ cup | 200 ml | 7 fl oz |  | *200 ml* | *6.95 g* |  |

Protein information obtained from: <https://ndb.nal.usda.gov/ndb/search/list>

***** The protein content of milk powder was calculated using: Basic Report:  01091, Milk, dry, non-fat, regular, without added vitamin A and vitamin D

****** The protein content of milk was calculated using: Basic Report:  01085, Milk, non-fat, fluid, with added vitamin A and vitamin D (fat-free or skim)

To convert g of milk to ml of milk a conversion of 1.031 was used i.e. 1 g milk = 103.1 g = 100 ml

******* The protein content of cheese was calculated using: Basic Report:  01009, Cheese, cheddar

**1.** Leonard SA, Nowak-Wegrzyn AH. Baked Milk and Egg Diets for Milk and Egg Allergy Management. Immunol Allergy Clin North Am. 2016;36(1):147-59.
